# Supplementary material for: Modeling glioblastoma heterogeneity as a dynamic network of cell states
Source: Mol Syst Biol. 2021 Sep 16;17(9):e10105. doi: 10.15252/msb.202010105 (PMC8444284; doi:10.15252/msb.202010105)
Supplement: Supplementary file 6 — Source Data for Figure 5 [file MSB-17-e10105-s004.zip › Figure5A_sourcedata/GSEA_3017/nosigdb_stateA.GseaPreranked.1622795026485/ALLEN_113_ASTROL1FGFR3FOS.html]

Details for gene set ALLEN\_113\_ASTROL1FGFR3FOS[GSEA]

|  || Dataset | state53017 |
| Phenotype | NoPhenotypeAvailable |
| Upregulated in class | na\_neg |
| GeneSet | ALLEN\_113\_ASTROL1FGFR3FOS |
| Enrichment Score (ES) | -0.648561 |
| Normalized Enrichment Score (NES) | -2.5019634 |
| Nominal p-value | 0.0 |
| FDR q-value | 1.0142858E-4 |
| FWER p-Value | 0.002 |
Table: GSEA Results Summary

  

Fig 1: Enrichment plot: ALLEN\_113\_ASTROL1FGFR3FOS      
 Profile of the Running ES Score & Positions of GeneSet Members on the Rank Ordered List

  

| PROBE | GENE SYMBOL | GENE\_TITLE | RANK IN GENE LIST | RANK METRIC SCORE | RUNNING ES | CORE ENRICHMENT || 1 | DBI |  |  | 17 | 0.683 | 0.0382 | No |
| 2 | KANK1 |  |  | 132 | 0.419 | -0.0451 | No |
| 3 | ATP13A4 |  |  | 410 | 0.290 | -0.3067 | No |
| 4 | ZFP36L1 |  |  | 532 | 0.264 | -0.4098 | No |
| 5 | S100B |  |  | 696 | -0.299 | -0.5533 | No |
| 6 | ID2 |  |  | 727 | -0.323 | -0.5579 | No |
| 7 | PON2 |  |  | 743 | -0.338 | -0.5458 | No |
| 8 | MEIS2 |  |  | 754 | -0.345 | -0.5279 | No |
| 9 | SLC4A4 |  |  | 763 | -0.355 | -0.5072 | No |
| 10 | NKAIN3 |  |  | 808 | -0.389 | -0.5209 | No |
| 11 | CST3 |  |  | 933 | -0.680 | -0.5931 | Yes |
| 12 | GLIS3 |  |  | 948 | -0.767 | -0.5450 | Yes |
| 13 | ADGRV1 |  |  | 950 | -0.771 | -0.4831 | Yes |
| 14 | CRYAB |  |  | 967 | -0.940 | -0.4229 | Yes |
| 15 | SLC1A3 |  |  | 973 | -1.097 | -0.3386 | Yes |
| 16 | NEAT1 |  |  | 986 | -2.049 | -0.1839 | Yes |
| 17 | GFAP |  |  | 987 | -2.255 | 0.0000 | Yes |
Table: GSEA details [plain text format]

  

Fig 2: ALLEN\_113\_ASTROL1FGFR3FOS: Random ES distribution      
 Gene set null distribution of ES for **ALLEN\_113\_ASTROL1FGFR3FOS**
